# Micronutrient Malnutrition in India: Let Us Say “No” to it Now

**DOI:** 10.4103/0970-0218.39235

**Published:** 2008-01

**Authors:** Prakash V Kotecha

**Affiliations:** A2Z Micronutrient Project, Academy for Education Development, 2 C-2 Parkwood Apartment, Raotula Ram Marg, New Delhi - 110 022, India

India's life expectancy has more than doubled, and infant mortality, halved in the last fifty years.(1) The extent of progress on economic fronts and political scenario has been enormous. India is knocking the door for a permanent position in the United Nations and is one of the few atomic power countries in the world. However, paradoxically, we have the highest number of malnourished people in India and our child malnutrition rate is unacceptably high. With one sixth of the global population residing in India, one third of about two billion people suffering from vitamin and micronutrient deficit are in India.

Micronutrients are required in small quantities and responsible for vital functions of the human body. Logically, they should be addressed easily and on a priority basis. The facts are, however, contrary. As a result, micronutrient malnutrition has been a persistent problem in India, and as the recent data suggest, some forms of micronutrient malnutrition are reaching their peak in the present century. We are broadly looking at the magnitude of this problem, and the initiatives taken by the government to tackle it and the results obtained with those efforts. Then, an effort is made to consider newer options and commitments required that are available for tackling the problem of micronutrient malnutrition.

The intake of micronutrients in daily diet is far from satisfactory and largely less than 50% RDA is consumed by over 70% of Indian population.(2) The loss due to micronutrient deficiency costs India 1 percent of its GDP. This amounts to a loss of Rs. 27,720 crore per annum in terms of productivity, illness, increased health care costs and death.

Every day, more than 6,000 children below the age of five die in India. More than half of these deaths are caused by malnutrition-mainly the lack of Vitamin A, iron, iodine, zinc and folic acid. About 57% of preschoolers and their mothers have subclinical Vitamin A deficiency.(3) Anemia prevalence among children under five years is 69% and among women it is over 55% in a recently concluded national study.(4) With the scientific reality of anemia being a late result of iron deficiency, these data reflect an almost universal iron deficiency in Indian population.

The consequences of micronutrient malnutrition are unacceptably high morbidity and mortality.(2) Vitamin A, iron and zinc deficiency when combined constitute the second largest risk factor in the global burden of diseases; 330,000 child deaths are precipitated every year in India due to vitamin A deficiency; 22,000 people, mainly pregnant women, die every year in India from severe anemia; 6.6 million children are born mentally impaired every year in India due to iodine deficiency; intellectual capacity is reduced by 15 per cent across India due to iodine deficiency; and 200,000 babies are born every year with neural tube defects in India due to folic acid deficiency.

Micronutrient malnutrition needs effective control on a priority basis. For effective control program for any disease, there are three essential components: a well defined goal, enabling policy and effective strategy.(5) These three components in turn need to be supported by Research and Development, Communication and Program Operations. Let us see how did India do in these regards for micronutrient malnutrition problem.

In the 10^th^ Five Year Plan (2002-2007),(6) the government of India set the following goals:

1. Eliminate Vitamin A deficiency as a public health problem.
2. Reduce the prevalence of anemia by 25%, and moderate and severe anemia by 50% in children, adolescents, pregnant and lactating women.
3. Achieve universal access to iodized salt.
4. Reduce the prevalence of IDD in the country to less than 10% by 2010.

These targets indicate a well-defined goal and policy planning by the government. However, the effective strategy to successfully achieve what is to be done was lacking. A program that did not have sound plan for implementation and that has a poor monitoring potential is likely to fail. Thus, a program that perhaps did not do well before 2002 as there were no clear goals set and program guidelines not worked out, continued to fail despite clear goals as the strategy did not specify who would do what and did not involve contingency planning. A strong monitoring component backed up by corrective action is required for positive results. Among the competing priorities of communicable diseases and eradication targeted programs such as that of poliomyelitis, one program that always got pushed back was that of micronutrient malnutrition. Thus during the 10^th^ five year plan, anemia prevalence increased instead of reduction as envisaged in the goals set by the five year plan.

It is with this reason largely that despite India being one of the first countries to start anemia control program along with initiating vitamin A supplementation, both as early as in 1970, the current scenario is not satisfactory. For almost two years now all the states are depleted of iron and folic acid tablet stock for pregnant women acknowledging that anemia contributes to 20% of maternal deaths every year in India. How can the Ministry of Health in the state department endorse this on one side and fail to utilize even 50% of funds made available to them under the National Rural Health Mission on the other?

The government is now committed to prioritize and work toward resolving micronutrient malnutrition. Indian Micronutrient Investment Plan(7) for 2007–2011 has been proposed by the Micronutrient Initiative, an international nongovernment organization working in collaboration with the Government of India.

In summary, the additional cost for control of micronutrient malnutrition works out to be Rs. 5.40 per capita per year, and Rs. 28.50 per high risk beneficiary. This additional cost constitutes 1% of the estimated per capital GDP loss incurred due to micronutrient malnutrition and 0.1% of the government expenditure. The total cost is thus about Rs. 77.6 crore. This is small amount as compared to budget of Rs. 3315 crore for ICDS expenses.

The challenges are high, but the promises are far reaching. We as public health specialists should refuse to accept these levels of micronutrient malnutrition and discourage set-ups where for two years at a primary health care level, iron and folic acid tablets are not made available to a severely anemic pregnant mother!
